# Supplementary material for: Intestinal microbes: an axis of functional diversity among large marine consumers
Source: Proc Biol Sci. 2020 Apr 1;287(1924):20192367. doi: 10.1098/rspb.2019.2367 (PMC7209056; doi:10.1098/rspb.2019.2367)
Supplement: Electronic Supplementary file [file rspb20192367supp1.pdf]

Intestinal microbes: an axis of functional diversity among large marine consumers

Electronic Supplementary Material

Jarrod J. Scott[a,1], Thomas C. Adam[b], Alain Duran[c], Deron E. Burkepile[b,d], & Douglas B. Rasher[e,1]

[a]Smithsonian Tropical Research Institute, APO 0843-03092 Balboa, República de Panamá
[b]Marine Science Institute, University of California, Santa Barbara, CA 93106, USA
[c]Dept. of Biological Sciences, Florida International University, Miami, FL 33199, USA
[d]Dept. of Ecology & Evolution, University of California, Santa Barbara, CA 93106, USA
[e]Bigelow Laboratory for Ocean Sciences, East Boothbay, ME 04544, USA
[1]To whom correspondence should be addressed. jarrod.jude.scott@gmail.com or drasher@bigelow.org

Contents

Content Description . . . 1
Data Availability . . . 2
Sample Naming . . . 2
Supplementary Methods . . . 2
Table S1 . . . 4
Table S2 . . . 4
Table S3 . . . 5
Table S4 . . . 5
Table S5 . . . 6
Table S6 . . . 6
Table S7 . . . 7
Figure S1 . . . 7
References . . . 8

This document contains supplementary tables and figures for the manuscript. In most cases, the tables were too large to fit in this document and are instead provided as additional tab-delimited text files. Regardless, below there are full descriptions for each table and figure.

If you prefer an interactive, HTML version of this file that includes all supplementary tables in a **single document**, you can download the file (https://doi.org/10.6084/m9.figshare.7379597) or view it online (https://projectdigest.github.io/supplemental_material.html).

Content Description

Data Availability: DOI and accession numbers for study related data.
Sample Naming: Naming scheme for samples.
Supplementary Methods: Details on DNA extraction, sequencing, and read processing, and functional analysis.
Table S1: Number of bites observed for each herbivore species at each site.
Table S2: Summary of field-based feeding observations.
Table S3: Metadata and microbiome diversity estimates for each sample.
Table S4: Total taxonomic diversity (by Class) of herbivore microbiomes.

Table S5: Results of LEfSe analysis.
Table S6: Results of BLAST analysis for DA ASVs.
Table S7: Accession numbers and unique codes for sequence data in Figure 3.
Figure S1: Class-level relative abundance of microbial communities from each sample.

Data Availability

We made additional data products and processing scripts available through online repositories.

- (https://doi.org/10.6084/m9.figshare.6875522): Raw data for each sample (before removing primers).
- (https://www.ebi.ac.uk/ena/data/view/PRJEB28397): Study accession number (PRJEB28397) for trimmed data (primers removed) deposited at the European Nucleotide Archive.
- (https://doi.org/10.6084/m9.figshare.6997253): DADA2 workflow for processing 16S rRNA reads.
- (https://doi.org/10.6084/m9.figshare.7357178): Reproducible phyloseq workflow. This includes the output from the DADA2 workflow, the phyloseq script, and other necessary input files.
- (https://doi.org/10.6084/m9.figshare.7379930): Data products from the workflows including otu_table, tax_table, sample_data table and ASV fasta files.
- (https://doi.org/10.6084/m9.figshare.7379936): Fasta file for the 59 DA ASVs, BLAST results, and alignment file including top BLAST hits.
- (https://doi.org/10.6084/m9.figshare.7379597): HTML version of this file.
- (https://projectdigest.github.io/): Project website that contains all the workflows and analyses.
- You can also access the complete figshare project repository here.

Sample Naming

Raw fastq data files were named using the root format *RunQ_GnSpe000_G*, where Q was the run number (1, 2, or 3), GnSpe was the host genus and species, 00 was a unique host ID number, and G was the gut segment (F = foregut; M = midgut; H = hind). For example, the file name *Run1_SpVir11_M_S147_L001_R2_001.fastq* corresponded to: the reverse read; midgut sample; *Sparisoma viride*; individual 11; Run01. All raw data are publicly available. The 53 individual fish encompassed seven species and three genera. Two species—*Sparisoma chrysopterum* and *Scarus vetula*—were only represented by 1 and 2 individuals, respectively. Though we omitted these samples from the analysis due to low sample size, they were sequenced and processed along with the rest of the samples. The data are publicly available.

Supplementary Methods

Study organisms

The five species of herbivorous fishes in this study—Acanthurus coeruleus, Acanthurus tractus, Scarus taeniopterus, Sparisoma aurofrenatum, and Sparisoma viride—all feed on benthic algae, but each species feeds in a different way and targets different components of the algal assemblage. Sp. viride is an excavating bioeroder that uses its strong, beak-like jaws to remove reef carbonates while feeding on endolithic algae [1]. The grazer Sc. taeniopterus primarily scrapes epilithic algal turfs from reef surfaces, while Sp. aurofrenatum tends to browse on erect macroalgae and longer turf algae that it tears from the reef [2,3]. Both A. coeruleus

and A. tractus crop algal filaments and browse on macroalgae, but the two species feed at different rates, digest food via different mechanisms [4], and display species-specific feeding preferences [5].

DNA extraction, sequencing, & read processing

For all samples, we homogenized material from each gut segment (fore, mid, hind) in separate 50 mL conical tubes for 10 minutes on a Vortex Genie 2. We collected 200 mg (wet weight) of homogenate for DNA extraction following the Human Microbiome Project Core Microbiome Sampling Protocol A (v12.0, HMP Protocol # 07-001) for stool samples. We heat treated each sample, first at 65°C for 10 minutes, followed by 95°C for 10 minutes. We then used the PowerSoil® DNA Isolation Kit (formerly MoBio, Carlsbad CA, USA) following the manufacturer's protocol to extract community DNA from each sample. Extracted DNA was sequenced on an Illumina MiSeq by Integrated Microbiome Resource (Dalhousie University). We targeted the V4–V5 hypervariable region using 515F and 926R primers [6]. We generated sequence data for 159 samples—three gut segments (fore, mid, and hind) from 53 individuals. Sequencing was conducted across three runs. In Run01, 144 samples were sequenced and, due to lower than average yield, were re-sequenced (Run02). The remaining 15 samples were sequenced on a separate run (Run03).

Cutadapt [7] was used to remove adapter sequences (max error rate = 0.12) and then mothur [8] was used to merge fastq files for each gut segment by individual and run (i.e., to pool the sequencing data produced among the three gut segments within an individual). We used DADA2 [9] following the Bioconductor workflow (v2) proposed by Callahan and colleagues [10] to filter and trim based on the quality profiles (maxN = 0; maxEE = 2, 5; truncQ = 2; and truncLen = 270, 200), error correct, dereplicate, and infer amplicon sequence variants (ASVs). ASVs are analogous to OTUs, but have higher (single nucleotide) resolution [11]. We merged pair-end reads, constructed sequence tables for each run, and removed amplicons > 380 or < 368 base pairs. We combined sequence tables from replicate runs (Run01 & Run02) and merged this table with the sequence table from Run03. Finally, we removed chimeras (method = "consensus") and assigned taxonomy against the Silva_nr_v132_train_set. See the electronic supplementary materials for details about obtaining the DADA2 workflow and associated data products.

In addition to the host species listed in the main text, we also collected two individuals from *Scarus vetula*, and one individual from *Sparisoma chrysopterum* in July 2016 at Pickles Reef, Upper Florida Keys, USA. Though these samples were sequenced and the data is publically available, the samples were removed from the study due to a low sample size of collected fish.

See the Data Availability section above for accession numbers and more details on obtaining raw sequencing data, workflows, data products, etc.

Inferring metagenomic function

We attempted to use PICRUSt and PICRUSt2 to predict the putative metagenome functional content of each microbiome; however, because so few intestinal microbiome studies have been conducted to-date with regard to herbivorous coral reef fishes, our weighted Nearest Sequenced Taxon Index (NSTI) scores were above the recommended cutoff (0.03). NSTI scores for PICRUSt ranged from 0.040 to 0.248 (mean: 0.099). PICRUSt2 performed slightly better with NSTI values from 0.045 to 0.166 (mean: 0.099), however still above the recommended cutoff. Therefore, we concluded that both PICRUSt nor PICRUSt2 were inappropriate analyses for these data.

See the PICRUSt paper (https://www.nature.com/articles/nbt.2676) and PICRUSt2 paper (https://www.biorxiv.org/content/10.1101/672295v1) for more details on these tools.

Additional alpha diversity tests

We also conducted Shapiro-Wilk Normality Tests on of the following alpha diversity metrics: Observed AVS, Chao1, and Inverse Simpson. Though only the Shannon index was normally distributed, we conducted additional tests of the non-normally distributed indices. Since host species is categorical, we used Kruskal-Wallis (non-parametric equivalent of ANOVA) to test for significance. For the inverse Simpson, Chao1, and

Observed richness the results of the Kruskal-Wallis rank sum test were all significant. We then used Wilcoxon rank sum test for post-hoc analysis of each pairwise comparisons for each index.

Again we saw that only *Sp. aurofrenatum* was significantly different from the other hosts. For the inverse Simpson index, *Sp. aurofrenatum* was significantly different from three of the four host species. For both Chao1 richness estimator and Observed ASV richness, *Sp. aurofrenatum* was significantly different from all other host species.

Complete code and results can be found on the project website.

https://projectdigest.github.io/4_diversity.html#statistical_tests

Associations between intestinal microbes, host phylogeny, & herbivore foraging behaviour

We used a series of simple and partial Mantel tests to assess whether DA ASVs were associated with a fish species' foraging ecology and/or phylogenetic history. The dissimilarity matrix for foraging ecology was based on the behavioural foraging data collected on the reef (see above). The phylogenetic dissimilarity matrix was based on a phylogenetic tree we constructed using cytochrome oxidase subunit 1 (COI) genes retrieved from NCBI's Nucleotide Database for the five fish species used in this study.

We used Clustal Omega [12] to align sequences (default settings for DNA) and Jalview [13] to manually curate and trim the final alignment to 593 bp. The alignment contained COI genes from *Sc. taeniopterus* (n = 5), *Sp. aurofrenatum* (n = 22), *Sp. viride* (n = 21), *A. coeruleus* (n = 28), and *A. tractus* (*bahianus*; n = 23), with members of the Gerridae (2 *Eucinostomus* and 4 *Gerres*) used as the outgroup. We used RAxML [14] and the GTR model for tree computation and the GAMMA rate model for likelihoods. The tree was then transformed into a distance matrix using the cophenetic function in R [15]. Finally, we constructed separate dissimilarity matrices to focus on putative resident ASVs and putative environmental ASVs, respectively. All matrices were constructed based on Bray-Curtis dissimilarity of Hellinger transformed data using the vegan package [16] in R [15].

Table S1

Table summarizing the number of individual bites observed by each of the five species of herbivorous fishes at each of three sites in the Florida Keys.

Host.species	Conch	French	Molasses	Total
Acanthurus coeruleus	118	47	114	279
Acanthurus tractus	121	47	123	291
Scarus taeniopterus	16	34	41	91
Sparisoma aurofrenatum	102	59	73	234
Sparisoma viride	83	71	65	219

Table S2

*This table is large and included as separate tab delimited text file (**Table_S2.txt**)*

Summary data from observations of individual bites by five species of herbivorous fishes in the Florida Keys. Data includes the mean sediment depth and turf height of algal assemblages fed on by each species at each of three sites, as well as the proportion of bites that resulted in a grazing scar on the substrate where reef calcium carbonate had been removed (Prop. grazing scar). Table summarizes the proportion of bites on vertical (> 45 degrees), concave, and convex substrates as well as the relative proportion of bites targeting each of the 10 food types most commonly bitten during the observations. Most macroalgae were aggregated to genus while other food types are summarized by functional group.

Table S3

*This table is large and included as separate tab delimited text file (**Table_S3.txt**)*

Table summarizing metadata for each host species and sample (**sample_ID**) used in this study. Includes various details about the host (e.g., weight, length, gut length, etc.) and the associated microbiome (e.g., number of reads, diversity stats, etc). Rows correspond to sample ID and columns correspond to various traits for each sample.

Rows correspond to sample ID and columns correspond to various traits for each sample. Below the table are column descriptions—though most data types are self-explanatory.

Column descriptions

Host details

- **SampleID**
- **Host genus**
- **Host species**
- **Common name**
- **NCBI tAxID** NCBI Taxonomic ID of host species.
- **Collection date**

Host physiological characteristics

- **Life phase**
- **Weight (g)**
- **Total length (cm)**
- **Foregut length (cm)**
- **Midgut length (cm)**
- **Hindgut length (cm)**
- **Total gut length (cm)**

Microbial diversity

- **Total reads** Total reads for each sample.
- **Total ASVs** Number of ASVs detected in each sample.
- **Chao1** The Chao1 richness estimator.
- **Chao1 (se)** Standard error of Chao1 index
- **ACE** The ACE richness estimator.
- **ACE (se)** Standard error of ACE index
- **Shannon** The Shannon diversity index
- **Simpson** The Simpson diversity index.
- **InvSimpson** Inverse Simpson's Index
- **Fisher** Fisher diversity Index.

Table S4

*This table is large and included as separate tab delimited text file (**Table_S4.txt**)*

Table showing the Class-level taxonomic diversity of the total microbiome dataset. Data shows the total number of reads and total number of ASVs for each Class plus the relative percent of each in the dataset.

Table S5

*This table is large and included as separate tab delimited text file (**Table_S5.txt**)*

Table showing the results from the LEfSe analysis including Linear discriminant analysis (LDA) scores, P-values adjusted for multiple testing, and False Discovery Rate (FDR) values. Normalized read abundance values (the input for the analysis) for each host species are also given.

Host abbreviations

- AcCoe: *Acanthurus coeruleus*
- AcTra: *Acanthurus tractus*
- ScTae: *Scarus taeniopterus*
- SpAur: *Sparisoma aurofrenatum*
- SpVir: *Sparisoma viride*

Table S6

*This table is large and included as separate tab delimited text file (**Table_S6.txt**)*

Full summary table of BLAST analysis for each of the 59 differentially abundant (DA) ASV. The table includes some details about each ASV (e.g., total reads), details of top BLAST hit(s), and alignment results. The table also includes results from queries to the IMNGS database.

Abbreviations

ND Indicates *No Data* was provided for given attribute of top BLAST hit.
NR In *Top hit acc* column indicates *Not Recorded*. Four ASVs (ASV6, ASV12, ASV224, ASV398) had numerous hits at 100% identity. We did not include *top hit* data for these ASVs.
NLC No Lifestyle Category from Sullam classification.

Column descriptions

ASV details

- **ASV** Query ASV.
- **Putative habitat** Proposed habitat preference based on data synthesis.
- **Enriched** Host species where ASV was differentially abundant.
- **Total reads** Total reads for query ASV in full dataset.
- **Taxon** Class-level taxonomic affiliation of query ASV.
- **Num IMNGS hits**. Number of hits to the IMNGS database. Value indicates the number of samples that scored a hit to an ASV based on 97% cutoff identity and representing greater than or equal to 0.1% of total reads in a sample from the database.

Details for top BLAST hits

- **Num perfect hits** Number of identical matches of query ASV to *nr* database (out of 50).
- **Top hit acc** Accession number of top BLAST hit. In cases of identical percent identity, all top hits are provided.
- **% identity** Percent identity of query ASV to top BLAST hit.
- **Isolation source** Tissue type of host (where applicable) where top BLAST hit was isolated from.
- **Nat host** Scientific name of host (where applicable) where top BLAST hit was isolated from.
- **Common name** Common name of host (where applicable) where top BLAST hit was isolated from.
- **Collection year** Year sample was collected (where provided) containing top BLAST hit.
- **Country** Country and location origin of sample (where provided) containing top BLAST hit.
- **PubMed ID** PubMed publication ID (where provided) containing top BLAST hit.

- **Sullam Lifestyle** Lifestyle classification of BLAST hit (where applicable) from the Sullam et. al. paper, specifically **Table S1**.

Alignment details between query ASV and top BLAST hit

- **Alignment length** Alignment length between query and subject.
- **Mismatches** Number of base pair mismatches in alignment between query and subject.
- **Gap opens** Number of open gaps in alignment between query and subject.
- **Q. start** Starting base pair position of query sequence in alignment.
- **Q. end** Ending base pair position of query sequence in alignment.
- **S. start** Starting base pair position of subject sequence in alignment.
- **S. end** Ending base pair position of subject sequence in alignment.
- **Evalue** The Expect (E) value of alignment.
- **Bit score** The bit score of alignment.

Table S7

*This table is large and included as separate tab delimited text file (**Table_S7.txt**)*

This table provides lookup details for all neighbor sequences from BLAST and SILVA-ACT comparisons used in the tree from Figure 3 (main paper). In the tree, sequences are named after the isolation source (e.g., *Naso unicornis*) plus a unique abbreviated code. This table gives the full accession numbers for each leaf.

- **Accession number** Full accession numbers for each leaf.
- **Tree code** Parenthetical code used in the tree.
- **Host, Isolation source, Location** Details about source of sequence.
- **Link** Hyperlink to sequence page from NCBI Nucleotide database.
- **Sullam lifestyle** In our analysis, we found the following categories: **1** *Vertebrate gut generalists*, **2** *Animal and vertebrate gut generalists*, **3** *Fish gut generalists*, **5** *Fish gut specialists*, **6** *Animal generalists*, **9** *Marine environmental microbes*, **12** *Microbes from artificial habitats*, **13** *Undefined, basal clade members.*

Figure S1

In Figure 2A of the main paper, Class-level abundance was displayed by host species. Here the same data is presented for each individual samples.

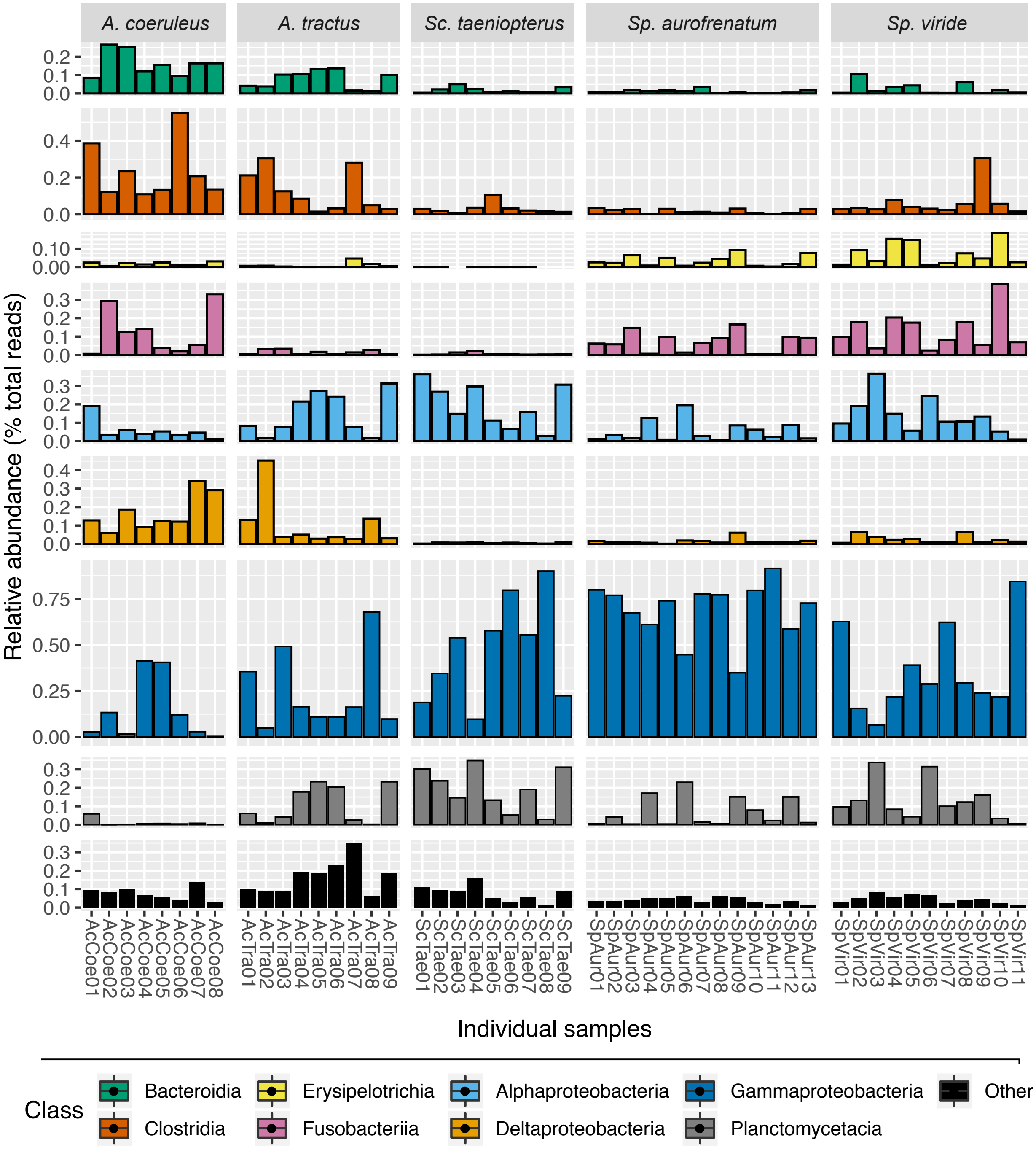

Supplementary Figure 1: *Class-level relative abundance of microbial communities from each sample. Taxa representing less than 2.5% total relative abundance were conglomerated into **Other**.*

References

1. Bruggemann JH, Kuyper MWM, Breeman AM. 1994 Comparative analysis of foraging and habitat use by the sympatric Caribbean parrotfish Scarus vetula and Sparisoma viride (Scaridae). Mar. Ecol. Prog. Ser. 112, 51–66.

2. Burkepile DE, Hay ME. 2008 Herbivore species richness and feeding complementarity affect community

structure and function on a coral reef. Proc. Natl. Acad. Sci. U. S. A. 105, 16201–16206.

3. Adam T, Duran A, Fuchs C, Roycroft M, Rojas M, Ruttenberg B, Burkepile D. 2018 Comparative analysis of foraging behavior and bite mechanics reveals complex functional diversity among Caribbean parrotfishes. Mar. Ecol. Prog. Ser. 597, 207–220.
4. Randall J. 1967 Food Habits of Reef Fishes of the West Indies. NOAA Rep.
5. Duran A, Adam TC, Palma L, Moreno S, Collado-Vides L, Burkepile DE. 2019. Feeding behavior in Caribbean surgeonfishes varies across fish size, algal abundance, and habitat characteristics. Mar. Ecol. 40 e12561.
6. Parada AE, Needham DM, Fuhrman JA. 2016 Every base matters: assessing small subunit rRNA primers for marine microbiomes with mock communities, time series and global field samples. Environ. Microbiol. 18, 1403–1414.
7. Martin M. 2011 Cutadapt removes adapter sequences from high-throughput sequencing reads. EMBnet.journal 17, 10.
8. Schloss PD et al. 2009 Introducing mothur: open-source, platform-independent, community-supported software for describing and comparing microbial communities. Appl. Environ. Microbiol. 75, 7537–7541.
9. Callahan BJ, McMurdie PJ, Rosen MJ, Han AW, Johnson AJA, Holmes SP. 2016 DADA2: high-resolution sample inference from Illumina amplicon data. Nat. Methods 13, 581–583.
10. Callahan BJ, Sankaran K, Fukuyama JA, McMurdie PJ, Holmes SP. 2016 Bioconductor workflow for microbiome data analysis: from raw reads to community analyses. F1000Research 5, 1492.
11. Eren AM, Zozaya M, Taylor CM, Dowd SE, Martin DH, Ferris MJ, 2011. Exploring the diversity of Gardnerella vaginalis in the genitourinary tract microbiota of monogamous couples through subtle nucleotide variation. PLoS One, 6 e26732.
12. Sievers F et al. 2011 Fast, scalable generation of high-quality protein multiple sequence alignments using Clustal Omega. Mol. Syst. Biol. 7, 539.
13. Waterhouse AM, Procter JB, Martin DMA, Clamp M, Barton GJ. 2009 Jalview Version 2–a multiple sequence alignment editor and analysis workbench. Bioinformatics 25, 1189–1191.
14. Stamatakis A. 2006 RAxML-VI-HPC: maximum likelihood-based phylogenetic analyses with thousands of taxa and mixed models. Bioinformatics 22, 2688–2690.
15. R Core Team. 2018 R: A Language and Environment for Statistical Computing.
16. Oksanen J et al. 2013 Package 'vegan'. Community Ecol. Packag. version 2.
